# Cuticular damage of *Lucilia cuprina* larvae exposed to *Curcuma longa* leaves essential oil and its major compound α-phellandrene

**DOI:** 10.1016/j.dib.2018.11.001

**Published:** 2018-11-06

**Authors:** Amanda Chaaban, Vinicius Sobrinho Richardi, Alessandra Regina Carrer, Juliana Sperotto Brum, Roger Raupp Cipriano, Carlos Eduardo Nogueira Martins, Mário Antônio Navarro-Silva, Cicero Deschamps, Marcelo Beltrão Molento

**Affiliations:** a Laboratory of Parasitic Diseases, Department of Veterinary Sciences, Federal University of Parana, UFPR, Curitiba, PR, Brazil; b Department of Veterinary Medicine, Catarinense Federal Institute, IFC, Araquari, SC, Brazil; c Laboratory of Morphology and Physiology of Culicidae and Chironomidae, Federal University of Parana, UFPR, Curitiba, PR, Brazil; d Laboratory of Veterinary Pathology, Department of Veterinary Sciences, Federal University of Parana, Curitiba, PR, Brazil; e Laboratory of Phytotech and Crop Protection, Federal University of Paraná, UFPR, Curitiba, PR, Brazil; f Agricultural Research and Extension of Santa Catarina, EPAGRI, Itajai, SC, Brazil; g National Institute of Science and Technology, INCT-Livestock, Belo Horizonte, MG, Brazil

## Abstract

Morphological biomarkers as the histopathological assessment and scanning electron microscopy can be used to establish a diagnosis of structure damage and intoxication of target cells by new biopesticide candidate. In this sense, cuticle damage caused by active substances in larvae exposed to biopesticides can help to elucidate the mode action. Thus, insecticide activity analysis of essential oil of *Curcuma longa* leaves and its major compound α-phellandrene have proven to be a new biopesticide candidate against third instar larvae (L3) of the Australian blowfly *Lucilia cuprina*. In this way, groups of 20 L3 were placed on filter paper, impregnated with ranging concentrations (from 0.15 to 2.86 μL/cm^2^) of *C. longa* leaves EO and (0.29–1.47 μL/cm^2^) to α-phellandrene. The extracts were solubilized in ethanol. Progressive darkening in the body of L3, marked reduction of movement, color changes in larval cuticle and dead were observed 6 and 24 h after contact with both extracts.

**Specifications table**

**Table:** 

Subject area	*Parasitology*
More specific subject area	*Entomology*
Type of data	*Videos*
How data were acquired	*Microscope stereoscopy*
Data format	*Raw data collection and analysis*
Experimental factors	*Fresh aerial parts of Curcuma longa (leaves) and its major compound α-phellandrene were assessed for insecticidal activity using biological assays on Lucilia cuprina performed as described by Chaaban et al., 2018*[1].
Experimental features	*Essential oil extraction and chemical characterization.*
	*Establishment of Lucilia cuprina colonies; and biological assays on laboratory conditions* (27 ± 2 °C and 70% *relative humidity*)*.*
	*Contact tests using filter paper impregnated with Curcuma longa leaves essential oil and its major compound α-phellandrene. Cuticular damage and larvae motility were reported.*
Data source location	*Araquari, Santa Catarina, Brazil; 26°23′ 33.6691″ S and 48° 44′ 18.3336″ W.*
Data accessibility	*Data are displayed within this article.*
Related research article	*This Data in Brief article is submitted as a companion paper to: Chaaban, A., Richardi, V.S., Carrer, Brum, J.S., Cipriano, R.R.,Martins, C.E.N., Silva, M.A.N., Deschamps, C., Molento, M.B. (in press)*. Insecticide activity of Curcuma longa (leaves) essential oil and its major compound α-phellandrene against Lucilia cuprina larvae (Diptera: Calliphoridae): Histological and ultrastructural biomarkers assessment. *Pesticide Biochemistry and Physiology*[1]

**Value of the data**

• Potential use of *Curcuma longa* and α-phellandrene as bioinsecticide against *Lucilia cuprina*.
• Contact activity of *C. longa* leaves essential oil and its major compound α-phellandrene over *L. cuprina* larvae.
• Determination of time-dependent damage of *L. cuprina* larvae exposed to *C. longa* leaves essential oil and α-phellandrene.

## 1 Data

The results of this study involve the experimental data from the cuticle damage of *L. cuprina* third instar larvae, exposed to *C. longa* leaves essential oil and its major compound α-phellandrene [1]. The larvae of the control group, using ethanol as solvent, showed no cuticle alterations after 6 and 24 h of contact (Video 1a, 1b; Video 2a, 2b). The insecticide effects of *C. longa* leaves EO and α-phellandrene can be observed ≤6 h after contact with the tested solutions (Video 1c; Video 2c). Moreover, progressive darkening in the body of L3, marked reduction of movement, color changes in larval cuticle and dead were observed in both extracts 24 h after exposure (Video 1d; Video 2d).

Supplementary material related to this data article can be found online at https://doi.org/10.1016/j.dib.2018.11.001.

The following is the Supplementary material related to this data article Video 1, Video 2.

Video 1 Third instar larvae (L3) of *L. cuprina*. (a) Control group (ethanol) 6 h after exposure. (b) Control group (ethanol) 24 h after exposure. Note normal size and movement and no change in cuticle color. (c) L3 treated with 1.59 µL/cm^2^ of *C. longa* leaves essential oil (CLLEO) 6 h after exposure. Observe the color change in larval body, decrease motility and some larvae dead. (d) L3 treated with 1.59 µL/cm^2^ of CLLEO 24 h after exposure. Note: progressive darkening in the body of L3 and marked reduction of movement

Video 2 Third instar larvae (L3) of *L. cuprina*. (a) Control group (ethanol) 6 h after exposure. (b) Control group (ethanol) 24 h after exposure. Note normal size and movement and no change in cuticle color. (c) L3 treated with 1.47 µL/cm^2^ of α-phellandrene 6 h after exposure. Observe the change in color of the anterior of L3 end and decrease motility. (d) L3 treated with 1.47 µL/cm^2^ of α-phellandrene 24 h after exposure. Note: accentuating darkening on dead larvae and recovery of motion in some larvae.

## 2 Experimental design, materials and methods

### 2.1 Plant material, essential oil extraction and chemical characterization

*C. longa* leaves used in this work were grown in the Medical Plants Unit of the Catarinense Federal Institute (IFC), located at 26° 23′ 33.6691″ S and 48° 44′ 18.3336″ W at 10.6 m above the sea level in the city of Araquari, Santa Catarina State, South of Brazil. The plant cultivation, essential oil extraction and chemical characterization were carried as described in the companion paper [1]. The α-phellandrene (CAS: 99-83-2) studied was acquired commercially and certified as having purity of ≥99%, from Sigma-Aldrich Brazil Ltda (São Paulo, SP, Brazil).

### 2.2 Establishment of *L. cuprina* colonies and larval toxicity

Wild flies were collected manually at the IFC, using bait and insect nets. The establishment of stock colonies, insects’ identification, maintenance, mass reproduction and the protocol for the biological tests were performed as described by Chaaban et al. [2]. The toxicity of *C. longa* leaves EO and α-phellandrene to *L. cuprina* larvae was performed using groups of 20 L3, placed on filter paper, impregnated with a range of concentrations (0.15–2.86 µL/cm^2^) of *C. longa* leaves EO and (0.29–1.47 µL/cm^2^) to α-phellandrene. The L3 were put into glass vials containing a filter paper (12.56 cm^2^) impregnated with 0.2 mL of solutions with EO, that were solubilized in ethanol. The toxicity was evaluated by observing L3 mortality at 6, 24 and 48 h after contact [1], [2]. Total larval mortality (LM) was calculated [1], [2], [3] as follows:

LM=(totaldiedlarvae×100)/totaltestedlarvae

For reported of the cuticle damage through the videos, L3 exposed to 1.59 µL/cm^2^ of *C. longa* leaves EO and 1.47 µL/cm^2^ of α-phellandrene solubilized in ethanol were used as described in the companion paper [1].

## Footnotes

Transparency document Transparency data associated with this article can be found in the online version at https://doi.org/10.1016/j.dib.2018.11.001.
