# Supplementary material for: Cuticular damage of Lucilia cuprina larvae exposed to Curcuma longa leaves essential oil and its major compound α-phellandrene
Source: Data Brief. 2018 Nov 6;21:1776–8. doi: 10.1016/j.dib.2018.11.001 (PMC6251368; doi:10.1016/j.dib.2018.11.001)
Supplement: Supplementary file 3 — Supplementary material. [file mmc1.doc]

 Ministério da Educação

Universidade Federal do Paraná

Setor de Ciências Agrárias

Departamento de Medicina Veterinária

Laboratório de Doenças Parasitárias

Dear Editor,

We are submitting a Data in Brief entitled: **Cuticular damage of *Lucilia cuprina* larvae exposed to *Curcuma longa* leaves essential oil and its major compound *α-*phellandrene**, to be considered for publication as a companion paper to: Insecticide activity of Curcuma longa (leaves) essential oil and its major compound α-phellandrene against Lucilia cuprina larvae (Diptera: Calliphoridae): Histological and ultrastructural biomarkers assessment accept to Pesticide Biochemistry and Physiology.

**The authors have no conflicts of interest to disclose.**

Please address all correspondence concerning this manuscript to: [amanda.chaaban@ifc.edu.br](mailto:amanda.chaaban@ifc.edu.br) and molento@ufpr.br.

Thank you for your consideration.

Best regards,

Marcelo Beltrão Molento

DVM, PhD, Assoc. Prof.

Med. Vet. CRMV 2729, PhD, UFPR
